# Demographic and clinical features of pediatric uveitis and scleritis at a tertiary referral center in China

**DOI:** 10.1186/s12886-022-02404-z

**Published:** 2022-04-18

**Authors:** Nan Sun, Chunxi Wang, Wenrui Linghu, Xiaorong Li, Xiaomin Zhang

**Affiliations:** Tianjin Key Laboratory of Retinal Functions and Diseases, Tianjin Branch of National Clinical Research Center for Ocular Disease, Eye Institute and School of Optometry, Tianjin Medical University Eye Hospital, Tianjin, China

**Keywords:** Pediatric uveitis, Scleritis, Cataract surgery, Steroid-induced ocular hypertension, Juvenile idiopathic arthritis, China

## Abstract

**Background:**

To analyse demographic, clinical features, treatment and therapeutic outcomes of pediatric uveitis and scleritis patients.

**Subjects:**

The clinical records of pediatric uveitis and scleritis cases between January 2012 and December 2020 at a tertiary uveitis service center in Tianjin Medical University Eye Hospital (TMUEH) were reviewed.

**Results:**

In total, 209 patients (337 eyes) were included, 49.3% were male. The median onset age was 9.0 (IQR, 7.0–12.0) years. Chronic uveitis and scleritis accounted for 86.1%. Panuveitis (29.2%), anterior uveitis(29.2%), and intermediate uveitis (22.0%) were the most common presentations. The most common diagnoses were idiopathic (71.3%), JIA (8.1%), and infectious uveitis (4.8%). At baseline, 40.7% patients received oral corticosteroid therapy; during follow-up, corticosteroids (66.0%), disease-modifying antirheumatic drugs (61.2%), and biologic agents (35.4%) were the mainstay. Posterior synechia (26.1%) and cataracts (25.5%) were the most common complications. In acute cases, the median best corrected visual acuity (BCVA) was 0.99 (IQR, 0.5–1.0) at baseline and 0 (IQR, 0–0) at last follow-up; in chronic cases, the median BCVA improved from 1.09 (IQR, 0.5–2.0) to 0.27 (IQR, 0–0.5), with anterior chamber cell grade significantly declining. Ten eyes underwent cataract surgery during regular follow-up and achieved satisfactory long-term visual outcomes and decreased burden of immunosuppressants. The incidence of steroid-induced ocular hypertension was 41.0%, and children with frequent instillation of eyedrops were at high risk.

**Conclusions:**

Most cases were of chronic uveitis and scleritis requiring long-term systemic immunosuppressive therapies in pediatric uveitis and scleritis in China. Good management of complications is important for long-term prognosis.

## Introduction

Pediatric uveitis accounts for only 5%–10% of all cases of uveitis, but it is of particular interest due to its diagnostic and therapeutic challenges. The onset of uveitis in children is often insidious and is only discovered during routine screening [1]. Although the incidence rates of scleritis are less frequently in children than in the adult population, scleral inflammation can be associated with systemic disorders [2]. Early diagnosis and prompt treatment are important to reduce the risk of both ocular and systemic secondary complications.

Most studies published on the epidemiology of pediatric uveitis and scleritis in Asia are from India, Japan and Singapore [3–5]. However, the pattern of pediatric uveitis and scleritis in China may differ from that in other Asian countries due to racial diversity.

Cataracts are common and frequent complications of chronic uveitis. The challenges include controlling inflammation to stop cataract development, proper timing of surgical intervention, and reduction of postoperative complications [6]. Inserting an intraocular lens (IOL) is commonplace for adult uveitic eyes but remains controversial for pediatric uveitic cataracts [6].

We aimed to describe the pattern of pediatric uveitis and scleritis at a tertiary eye care center in North China and report our outcomes of cataract surgery with primary IOL implantation in a cohort of children with chronic uveitis.

## Methods

We retrospectively analysed the medical records of uveitis and scleritis patients who were diagnosed at 16 years of age or younger between January 2012 and December 2020 at a tertiary uveitis service at Tianjin Medical University Eye Hospital (TMUEH). Institutional review board approval (No. 2021KY (L)-10) from TMUEH was obtained for the retrospective review of clinical records of all patients.

Routine laboratory workup, including complete blood count, serum biochemical analysis including liver and kidney function indicators, erythrocyte sedimentation rate, and immunological indicators, purified protein derivative (PPD), and tests for syphilis, and human immunodeficiency virus (HIV) were performed. Immunological investigations included HLA-B27, antinuclear antibodies (ANA), antistreptolysin (ASO), erythrocyte sedimentation rate (ESR), C-reactive protein (CRP), extractable nuclear antigen (EDN), rheumatoid factor (RF), anti-neutrophil cytoplasmic antibodies (ANCA), and anticardiolipin antibodies (ACA). Further investigations were targeted as per history and examination; infectious aetiologies were detected through various tests. All patients suspected of having systemic disease were referred to a rheumatologist. The final diagnosis was made according to the International Uveitis Study Group classification [7]. Uveitis was classified using its anatomical location as anterior, intermediate, and posterior uveitis and panuveitis according to the standardised uveitis nomenclature guidelines [8]. The classification of anterior and posterior scleritis was based on the Watson and Hayreh classification [9]. We also classified patients according to their associated autoimmune systemic diseases [10–12]. If no identifiable causes were found, the term idiopathic uveitis was used.

Uveitis and scleritis was treated depending on its localization and course of the disease. For patients with inflammation in anterior chamber, topical corticosteroids and cycloplegic agents were used; for bilateral chronic anterior uveitis, unilateral or bilateral intermediate uveitis, posterior uveitis and panuveitis, systemic corticosteroids combine with disease-modifying antirheumatic drugs (DMARDs) were used for most of the cases. In refractory cases that were resistant to these therapies or when significant side effects occurred or the parents refused long-term of corticosteroid therapy, biologics agents were added. Hematological, hepatic, and renal functions were monitored regularly during follow-up [13, 14].

Data were extracted from medical records. Information on treatment were included in the registry. BCVA was measured using a Snellen chart. The equivalent logarithm of the minimum angle of resolution (logMAR) was calculated and used for analysis [15]. Complications included posterior synechiae, cataract, band keratopathy, uveitic ocular hypertension (uveitic OHT, defined as intraocular pressure > 21 mmHg without optic disk or visual field changes), steroid-induced ocular hypertension (SIOHT, defined as IOP increase > 5 mmHg due to glucocorticoid use in the eye) [16], uveitic glaucoma (UG, defined as repeated IOP measurements > 21 mmHg with optic disk changes and/or visual field changes), and cystoid macular edema (CME). Ocular surgery was classified according to the indication. To evaluate the evolution of chronic uveitis patients undergoing cataract surgery with IOL implantation, clinical data were collected. ACC (aqueous humor cell count) [7] and immunosuppression load [17] were graded as described previously.

The incidence of SIOHT was also investigated. We analysed a group of patients with these characteristics: lack of follow-up records or documented intraocular pressure (IOP), and elevation of IOP due to other related factors. Moreover, based on the Armaly and Becker classification [18], we categorised the SIOHT responses into high responders (IOP rise above 15 mmHg) and moderate responders (IOP rise between 6–15 mmHg).

Statistical analyses were performed using GraphPad Prism (GraphPad Prism 8.0.1; GraphPad Software, San Diego, CA, USA). Continuous variables are expressed as mean ± standard deviation (SD) or median (interquartile range, IQR). T-tests or ANOVA tests were performed to compare normally distributed data. The Mann–Whitney U test was used to compare abnormally distributed data. Statistical significance was set at *P* <  0.05.

## Results

### Patient demographics

The characteristics of all patients are summarised in Table 1. Overall, 209 patients (337 eyes) diagnosed at 16 years of age or younger with uveitis and scleritis were evaluated. A total of 103 patients (49.3%) were men, and 106 (50.7%) were women. The median onset age was 9.0 (IQR, 7.0–12.0) years, and the median baseline age was 11.0 (IQR, 8.0–14.0) years. The median time between onset and referral to our clinic was 5.0 (IQR, 1.0–19.0) months. The median follow-up was 3.8 (IQR, 0.5–13.0) months.

**Table 1:** Demographic features of all paediatric patients. (*N* = 209 patients)

		**Total**
Patients (eyes)		209 (337)
Sex	Male (n, %)	103 (49.3)
	Female (n, %)	106 (50.7)
Age	Median baseline age (years, IQR)	11.0 (8.0–14.0)
	Median onset age (years, IQR)	9.0 (7.0–12.0)
Referral time	Median referral time (months, IQR)	5.0 (1.0–19.0)
Follow-up time	Median follow-up (months, IQR)	3.8 (0.5–13.0)

### Uveitis and scleritis characteristics

Table 2 shows the anatomical and pathogenic distribution of the 209 patients. Uveitis and scleritis manifested as acute (29 patients, 13.9%) and chronic (180 patients, 86.1%) disease. Bilateral involvement was observed in 128 patients (61.2%). Anterior uveitis (61 patients, 29.2%) and panuveitis (61 patients, 29.2%) were the most common presentation, followed by intermediate uveitis (46 patients, 22.0%) and posterior uveitis (21 patients, 10.0%). Posterior scleritis was observed in 15 patients (7.2%), followed by nodular anterior scleritis (three patients, 1.4%), diffuse anterior scleritis (one patient, 0.5%), and episcleritis (one patient, 0.5%). With regard to different aetiologies, idiopathic (149 patients, 71.3%) was the most common diagnosis, followed by juvenile idiopathic arthritis (JIA, 17 patients, 8.1%), and infectious uveitis (10 patients, 4.8%). Thirty-three patients (15.8%) had systemic diseases, including JIA (17/33, 51.6%), VKH (7/33, 22.6%), Behçet's disease (4/33, 12.9%), Blau syndrome (2/33, 6.5%), masquerade syndromes (2/33, 6.5%), and multiple sclerosis (1/33, 3.0%). The types of JIA included oligoarthritis (nine patients), RF-positive polyarthritis (one patient), RF-negative polyarthritis (four patients), enthesitis-related arthritis (three patients), and ANA in five patients (three oligoarthritis, one RF-positive polyarthritis, and one RF-negative polyarthritis). Infectious uveitis included three cases of acute retinal necrosis (ARN) caused by type I herpes simplex virus (HSV), ocular toxoplasmosis in one case, ocular toxocariasis in three cases, one chronic anterior sclerouveitis case caused by varicella zoster virus (VZV), and two clinically diagnosed anterior uveitis with unconfirmed virus. Parameters relevant for immunology were retrieved for patients whose immunology results were available: 10 patients were positive for HLA-B27, including four cases of HLA-B27 related idiopathic uveitis, three cases of JIA, two cases of scleritis, and one case of VKH; 11 patients were positive for ANA, including five JIA-U patients: three oligoarthritis, one RF positive polyarthritis, and one RF negative polyarthritis; 10 patients were positive for ASO, including eight cases of idiopathic uveitis, one case of JIA and one case of Behçet's disease, and six patients experienced attacks during spring or winter; 10 patients were positive for ESR and five patients were positive for CRP.

**Table 2:** Distribution of uveitis and scleritis according to the different classification criteria. (*N* = 209 patients)

		**Anterior uveitis (n, %)**	**Intermediate uveitis (n, %)**	**Posterior uveitis (n, %)**	**Panuveitis (n, %)**	**Episcleritis (n, %)**	**Diffuse anterior scleritis (n, %)**	**Nodular anterior scleritis (n, %)**	**Posterior scleritis (n, %)**	**Number of patients**	**%**
**Bilaterality**		31 (24.2)	41 (32.0)	10 (7.8)	37 (28.9)	0	0	1 (0.8)	8 (6.3)	128	61.2
**Course**	Acute	24 (82.8)	0	0	4 (1.4)	1 (3.4)	0	0	0	29	13.9
	Chronic	36 (20)	47 (26.1)	22 (12.2)	57 (31.7)	0	1 (0.6)	2 (1.1)	15 (8.3)	180	86.1
Non-infectious uveitis	Idiopathic	28 (18.8)	41 (27.5)	16 (10.7)	44 (29.5)	1 (0.7)	1 (0.7)	3 (2.0)	15 (10.1)	149	71.3
	JIA	17 (100)	0	0	0	0	0	0	0	17	8.1
	VKH	0	0	0	7 (100)	0	0	0	0	7	3.3
	HLA-B27 related	0	4 (100)	0	0	0	0	0	0	4	1.9
	Posner-Schlossman Syndrome	6 (100)	0	0	0	0	0	0	0	6	2.9
	Behçet's	0	1 (25)	1 (25)	2 (50)	0	0	0	0	4	1.9
	Fuchs syndrome	4 (100)	0	0	0	0	0	0	0	4	1.9
	Blau syndrome	1 (50)	0	0	1 (50)	0	0	0	0	2	1.0
	Masquerade syndromes	2 (100)	0	0	0	0	0	0	0	2	1.0
	Multiple sclerosis	0	0	0	1 (100)	0	0	0	0	1	0.5
	TINU	1 (50)	0	0	1 (50)	0	0	0	0	2	1.0
	Phacoallergic uveitis	1 (100)	0	0	0	0	0	0	0	1	0.5
	Total	59 (29.6)	46 (23.1)	17 (8.5)	57 (28.6)	1 (0.5)	1 (0.5)	3 (1.5)	15 (7.5)	199	95.2
Infectious uveitis	ARN	0	0	0	3 (100)	0	0	0	0	3	1.4
	Toxoplasmosis	0	0	1 (100)	0	0	0	0	0	1	0.5
	Toxocariasis	0	0	3 (100)	0	0	0	0	0	3	1.4
	VZV	1 (100)	0	0	0	0	0	0	0	1	0.5
	Unknown	1 (50)	0	0	1 (50)	0	0	0	0	2	1.0
	Total	2 (20)	0	4 (40)	4 (40)	0	0	0	0	10	4.8
**Total**		61 (29.2)	46 (22)	21 (10.0)	61 (29.2)	1 (0.5)	1 (0.5)	3 (1.4)	15 (7.2)	209	100.0

*JIA* Juvenile idiopathic arthritis, *VKH* Vogt-Koyanagi-Harada, *TINU* Tubulu-interstitial nephritis-uveitis, acute retinal necrosis syndrome, *VZV* varicella zoster

### Medication use

Different lines of treatment before the visit and during follow-up, including corticosteroids, DMARDs, and biologics, were reported (summarised in Table 3). The ratio of patients using corticosteroid eye drops increased from 45.0% to 55.2% after visiting us; the ratio of oral corticosteroids increased from 40.7% to 66.0% after visiting us; the ratio of monotherapy decreased from 17.2% to 7.2%, while the ratio of combined therapy, including corticosteroids combined with non-biologic DMARDs and/or biologic agents, increased from 23.9% to 58.9%. Systemic DMARDs were used in 25.4% and 61.2% patients, and biologic agents were used in 5.7% and 35.4% patients respectively before and after visit. Adalimumab was the major biologics used after visit (69 patients, 33%). We observed that 117 patients did not receive systemic therapy before visit, and 53.8% (63/117) were administered at least one systemic medicine after the visit, including 23 cases of intermediate uveitis, 19 of panuveitis, 12 of anterior uveitis, five of posterior uveitis, and four of posterior scleritis.

**Table 3:** Different lines of treatment before visit and during follow-up (*N* = 209 patients)

		**Number of patients before visit**	**%**	**Number of patients during follow-up**	**%**
Corticosteroid eye drops		94	45.0	160	55.2
Oral corticosteroids		85	40.7	138	66.0
	Corticosteroid monotherapy	36	17.2	15	7.2
	Corticosteroid + DMARDs /biologic agent	50	23.9	123	58.9
DMARDs		53	25.4	128	61.2
	Methotrexate	20	9.6	97	46.4
	Ciclosporin A	40	19.1	70	33.5
	Mycophenolate mofetil/sodium	10	4.8	16	7.7
	Azathioprine	2	1.0	5	2.4
Biologic agent		12	5.7	74	35.4
	Adalimumab	6	2.9	69	33.0
	Infliximab	1	0.5	20	9.6
	Golimumab	3	1.4	1	0.5
	Etanercept	3	1.4	0	0.0
	Tocilizumab	0	0.0	1	0.5

### Complications and surgery

As shown in Table 4, ocular complications were observed in 176 (52.2%) of the 337 eyes. The most common complications were posterior synechia (88 eyes, 26.1%), followed by cataract (86 eyes, 25.5%), band keratopathy (33 eyes, 9.8%), cystoid macular edema (33 eyes, 9.8%), papilledema (32 eyes, 9.5%), ocular hypertension (14 eyes, 4.2%), and corneal edema (six eyes, 1.8%). Three eyes with ocular hypertension developed glaucoma. Most of the complications (93.8%) occurred before visiting the clinic due to previous inappropriate therapies, delayed visits, or misdiagnosis. Forty-seven eyes (13.9%) underwent ocular surgery. Phzacoemulsification and IOL implantation were performed in 39 eyes (11.6%), iris YAG laser drilling in ten eyes (3.0%), peripheral iris resection in nine eyes (2.7%), drainage valve implantation in three eyes (1.3%), PPV in four eyes (1.2%), ciliary body cryotherapy, trabeculectomy, and pupilloplasty in one eye (0.3%).

**Table 4:** Complications and surgery of uveitis and scleritis patients (*N* = 337 eyes)

		**Number of eyes**	**%**
Complications		176	52.2
	Posterior synechiae	88	26.1
	Cataract	86	25.5
	Band keratopathy	33	9.8
	Cystoid macular oedema	33	9.8
	Papilledema	32	9.5
	Retinal detachment	12	3.6
	Ocular hypertension	14	4.2
	Corneal oedema	6	1.8
	Eyeball atrophy	2	0.6
	Pupillary membrane closure	1	0.3
Surgery		47	13.9
	Phaco + IOL	39	11.6
	Iris YAG laser drilling	10	3.0
	Peripheral iris resection	9	2.7
	PPV	4	1.2
	Drainage valve implantation	3	0.9
	Ciliary body cryotherapy	1	0.3
	Trabeculectomy	1	0.3
	Pupilloplasty	1	0.3

Cataract occurred in 86 eyes (86/337, 25.5%) of 59 patients, while 251 eyes of 150 patients did not develop cataracts. We compared the clinical characteristics of eyes with cataract and those without cataracts. As shown in Table 5, the most common diagnosis was Fuchs syndrome (3/4, 75%), followed by JIA-associated uveitis (19/30, 63%), Blau syndrome (1/2, 50%), Behçet's disease (2/7, 28.6%) and VKH (4/14, 28.6%). Of 86 eyes complicated with cataract, 36.5% had anterior uveitis (34 eyes), 34.5% had panuveitis (34 eyes), 23.3% had posterior uveitis (seven eyes), 10% had scleritis (three eyes), and 9.3% had intermediate uveitis (eight eyes). Cataract occurred in 34.4% of the bilateral uveitis and scleritis patients (44 patients).

**Table 5:** Demographic and clinical characteristics of patients developing cataract. (*N* = 337 eyes)

		**Cataract** **(n, %)**	Total
Sex (per eye)	Male	35 (20.5)	171
	Female	51 (30.7)	166
Aetiology (per eye)	Idiopathic uveitis	50 (23.1)	216
	JIA-U	19 (63.3)	30
	VKH	4 (28.6)	14
	HLA-B27 related	2 (25)	8
	Posner-Schlossman Syndrome	0	7
	Behçet' disease	2 (28.6)	7
	Fuchs syndrome	3 (75.0)	4
	Blau syndrome	2 (50)	4
	Masquerade syndromes	0	2
	Infectious uveitis	0	12
	Scleritis	3 (15)	30
	TINU	0	2
	Multiple sclerosis	1 (100)	1
Anatomy (per eye)	Anterior	34 (36.5)	93
	Intermediate	8 (9.3)	86
	Posterior	7 (23.3)	30
	Panuveitis	34 (34.5)	98
	Scleritis	3 (10)	30
Laterality (per patient)	Unilateral	15 (18.5)	81
	Bilateral	44 (34.4)	128

Nineteen eyes (14 patients) underwent surgery for complicated cataracts. The median follow-up time was 13.4 (IQR, 4.2–47.2) months. Eight eyes had anterior uveitis (42.1%, six patients), two eyes (10.5%, one patient) with posterior uveitis, and nine eyes (47.4%, seven patients) with panuveitis, including eight cases of idiopathic uveitis, five cases of JIA, and one case of Blau syndrome. Nine patients (10 eyes) underwent cataract surgery in our hospital after rigorous inflammation control, with ‘zero ACC’ for at least three months. The median baseline age was 6.5 (IQR, 4.8–11.0) years, the median course before visit was 6.5 (IQR, 1.3–17.3) months, and the median evolution time before surgery was 14.9 (IQR, 5.7–26.2) months. The mean logMAR BCVA improved from 2.0 (SD: 1.1) to 0.3 (SD: 0.1) LogMAR 12 months after the operation (*p* = 0.0004), and the median ACC was graded 0.25 (IQR, 0–0.5) 12 months after the operation, and the burden of immunosuppression was successfully decreased from 8.1 (SD: 2.2) to 1.9 (SD: 1.8) (*p* = 0.01).

### Treatment effect

**Fig. 1:**
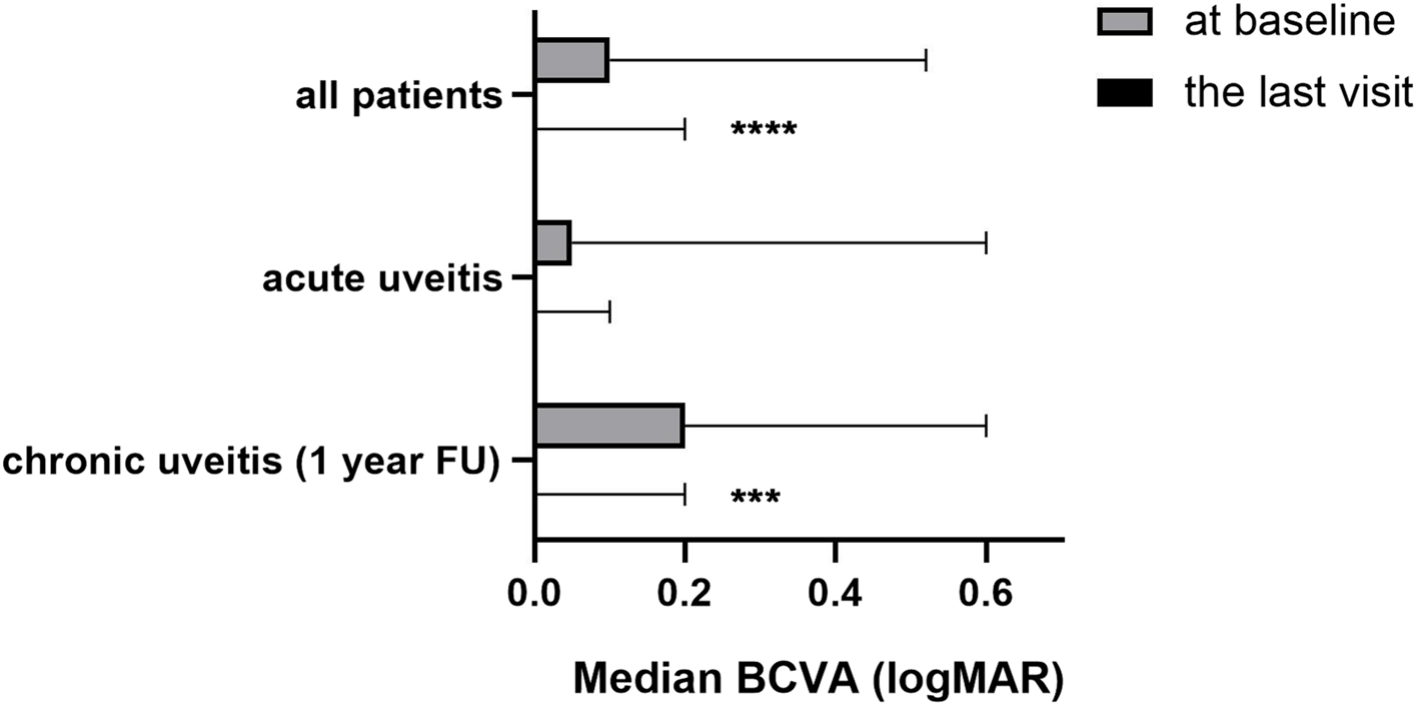
Median BCVA in eyes with all paediatric uveitis, acute uveitis, and chronic uveitis (1-year FU) at baseline and at the last visit. FU, final follow-up

The median ACC grade declined significantly from 1.0 (IQR, 0.5–2.0) to 0 (IQR, 0–0.5) in all eyes (*n* = 312, *p* = 0.000), from 0.99 (IQR, 0.5–1.0) to 0.0 (IQR, 0–0) in acute uveitis eyes, from 1.09 (IQR, 0.5–2.0) to 0.27 (IQR, 0–0.5) in eyes with chronic uveitis and scleritis who had at least a 1-year follow-up, respectively. As shown in Fig. 1, the median BCVA of eyes in all patients significantly improved from 0.1 (IQR, 0–0.52) LogMAR at baseline (*n* = 312) to 0 (IQR, 0–0.2) logMAR at the last visit (*n* = 246) (*p* < 0.0001); in eyes with acute cases (*n* = 27), the median BCVA improved from 0.05 (IQR, 0–0.6) logMAR at baseline to 0 [IQR, 0–0.1) logMAR at the last visit (*p* = 0.16), and the median BCVA improved from 0.2 (IQR, 0–0.6) logMAR to 0 (IQR, 0–0.2) logMAR in eyes with chronic cases with at least 1 year of follow-up (*n* = 95) (*p* = 0.0003).

### Steroid-induced ocular hypertension

A total of 134 patients (males accounted for 50.4%) who used local corticosteroid therapy and had at least one return visit were reviewed; SIOHT occurred in 55 children (41.0%), induced by 1% prednisolone acetate (40 patients), loteprednol etabonate eye drops (four patients), fluorometholone eye drops (two patients), dexamethasone eye drops (one patient), triamcinolone acetonide through parabulbar injections (four patients), and uncertain corticosteroid eye drops (four patients). As shown in Table 6, we analysed 93 patients using 1% prednisolone acetate alone with long-term follow-up (32 responders, 61 non-responders), and found no significant difference in sex, age, or treatment duration between responders and non-responders. Among 32 responders using 1% prednisolone acetate, the mean age was 10.0 (IQR, 7.3–13.0) years old, and baseline IOP ranged from 6.9 to 25.6 mmHg (mean 12.2 mmHg, SD: 3.7 mmHg) while response IOP ranged from 18.4 to 50.7 mmHg (median 26.6 mmHg, IQR 22.0–30.4). The median daily dose was 3.7 (IQR, 2.9–4.5) drops among responders, and the median duration of corticosteroid eye drops was 28.0 (IQR, 14.5–34.5] days). The median daily dose in non-responders was significantly lower than responders (3.0 VS 3.7 drops/day, *p* = 0.005). As shown in Table 7 data were analysed according to the duration of topical corticosteroid administration as less than 14 days, 14 days to 30 days, and more than one month. It’s interesting that among the responders who used 1% prednisolone acetate within 14 days, there were more high responders (66.7%).

**Table 6:** Clinical features of patients who used 1% prednisolone acetate eye drops. (*N* = 93 patients)

	**Responders**				**Non-responders**	***P* value** **(Responders vs Non-responders)**
	**Intermediate responders (IR)**	**High responders** **(HR)**	***P* value** (**IR vs HR)**	**Total**		
Number of patients	17	15	-	32	61	-
Male	4	7	0.13	11	31	0.19
IOP range	(6.9–18.0) to (18.4–28.8)	(7.5–25.6) to (26.2–50.7)	-	(6.9–25.6)—(18.4–50.7)	-	-
Age	10.0 ± 3.2	9.8 ± 3.8	0.91	10.0 (7.3–13.0)	11.0 (6.5–14.5)	0.62
Daily doses (drops/day)	3.7 (3.0–4.0)	4.0 (3.4–5.9)	0.25	3.7 (2.9–4.5)	3.0 (1.9–4.0)	0.005
Median use duration (days)	28.0 (16.8–35.3)	26.0 (14.0–39.0)	0.63	28.0 (14.5–34.5)	29.0 (18.5–58.0)	0.17

**Table 7:** Clinical features in ocular hypertension responders induced by 1% prednisolone acetate (*N* = 32 patients)

	**Use duration before SIOHT (days)**		
	** < 14d**	**14-30d**	** > 30d**
Number of patients (%)	6 (18.8)	15 (46.9)	11 (34.4)
High responders (%)	4 (66.7)	6 (40.0)	5 (45.5)
Male (%)	2 (33.3)	4 (26.7)	5 (45.5)
Age	10.7 ± 3.9	10.0 ± 4.0	10.3 ± 2.1
Daily doses (drops/day)	4.0 (3.6–6.0)	3.9 (2.9–4.5)	3.5 (3.1–4.2)
Magnitude of elevated IOP	18.0 (11.3–21.1)	13.0 (12.0–18.0)	14.0 (11.0–18.0)

*SIOHT* steroid-induced ocular hypertension

## Discussion

The present study specially reveals the characteristics of pediatric uveitis in China for the first time. Literature are summarised in Table 8, which report the characteristics of pediatric uveitis from tertiary uveitis clinics in Asia (Japan, Singapore, India, Egypt, Turkey, and Israel), North America (USA) and South America (Brazil) [3, 19–28]. Although most studies reported a female predominance, especially in developed countries (Japan, Singapore, and the USA), our study showed that the proportion of pediatric uveitis was similar between men (49.3%) and women (50.7%). The proportion of male patients increased to 52.6% when scleritis was excluded. The mean age at the onset of uveitis was 9.0 years, similar to most studies, except for reports from Israel, Egypt, and Brazil, which showed a younger onset age.

**Table 8:** The comparison of our study with different series about pediatric uveitis and scleritis reported from various regions

Country	Number of patients (Male, %)	Onset age	Course (%)	Infectious (%)	Non-infectious autoimmune aetiology (%)	Idiopathic (%)	Most common systemic disease (%)	Anatomical classification (%)	Complications (%)
Current Study	209 (49.3%)	9.0 (7.0–12.0)	Chronic (86.1%)	Uvietis (4.80%);	Uvietis (85.6%); Scleritis (9.6%)	63.20%	JIA (8.1%)	Anterior uveitis/Panuveitis (29.2%)	Cataract (25.5%); Posterior synechiae (26.1%)
Singapore 2020	73 (35.6%)	12.1 ± 3.0	Chronic: Uveitis (61.1%); Scleritis (100%)	Uveitis (33.3%); Scleritis (10.5%)	Uveitis (37.0%); Scleritis (89.5%)	Uveitis (29.6%); Scleritis (68.4%)	Uveitis (Sarcoidosis, 14.8%); Scleritis (Sarcoidosis/HLA-B27, 10.5%)	Uveitis (Posterior, 27.8%); Scleritis (Posterior scleritis, 94.7%)	Uveitis (Cataract, 40.7%); Scleritis (Glaucoma, 36.8%)
Turkey 2020	93 (43.0%)	9.54 ± 4.29	Chronic (49.5%)	12.9%	87.1%	43.0%	JIA (18.3%)	Anterior uveitis (49.5%)	Posterior synechiae (18.6% of 156 eyes)
Japan 2020	98 (34.7%)	11.4 ± 4.1	Unknown	Unknown	Unknown	35.70%	Juvenile chronic iridocyclitis (29.6%)	Anterior (52.0%)	Increased intraocular pressure (33.7%)
America 2019	49 (45%)	9.15 ± 4.2	Chronic (61.2%)	20.40%	79.60%	51.00%	JIA (12.2%)	Anterior (40.8%)	Vitreal haze (37.5% of 80 eyes); Posterior synechiae (32.5% of 80 eyes)
Turkey 2019	76 (52.6%)	9.5 ± 3.9	Unknown	Unknown	100%	50%	JIA (25%)	Intermediate (34.2%)	Glaucoma (7.7% of)
America 2019	118 (34%)	7.4	Unknown	Unknown	100%	33%	JIA (57%)	Anterior uveitis (82.0%)	Cataract (26%)
America 2018	286 (37.8%)	8.4 ± 3.83	Recurrent (68.53%)	3.50%	96.50%	51.40%	JIA (34.96%)	Anterior (61.9%)	Cataract (43.84% of 520 eyes)
Israel 2018	107 (45.0%)	8.8 ± 4.4	Chronic (65% of 182 eyes)	6.50%	93.50%	53.30%	JIA (25.2%)	Anterior uveitis (48.0%)	Posterior Synechiae (22.5% of 182 eyes)
Egypt 2018	413 (53%)	7.8 ± 2.9	Chronic (72.9%)	Unknown	Unknown	28.60%	Tuberculosis (13.1%); Sarcoidosis (12.8%)	Intermediate (30.0%)	Cataract (31.1%)
Brazil 2018	39 (35.9%)	6.3 ± 3.6	Recurrent/chronic (84.6%)	17.90%	82.10%	5.10%	JIA (41%)	Anterior (46%)	Cataract (19 eyes)
India 2016	190 (64.0)	11	unknown	23%	77%	37.40%	JIA (13%)	Anterior (52%)	Cataract (44%)

Anterior uveitis (29.2%) and panuveitis (29.2%) were the most common types, followed by intermediate uveitis (22.0%), which was consistent with other studies. Panuveitis was as common as anterior uveitis in our cohort. Infectious uveitis accounted for 4.8%, similar to recent reports (3.5%–20.4%). Idiopathic uveitis and scleritis prevailed in 71.3% of patients, which is higher than that in recent reports from other countries (5.1%–53.3%). At our center, idiopathic uveitis and scleritis is diagnosed clinically after the exclusion of systemic disease by detailed clinical history and routine laboratory workup at the first visit according to the International Uveitis Study Group [7]. The high proportion of idiopathic may be due to ethnic differences or the low detection rate of systemic diseases at the beginning of uveitis and scleritis in the primary hospital, which cannot be detected after systemic immunosuppressive therapy. JIA was diagnosed in 8.1% of our patients. Substantial disparities were observed between geographical regions in the prevalence of JIA-U. An international observational cohort study reported that the prevalence of JIA-U was the highest in northern Europe (19.1%) and southern Europe (18.8%) and lowest in Latin America (6.4%), Africa, the Middle East (5.9%), and Southeast Asia (5.0%) [29]. One previous study that focused on epidemiological characteristics of uveitis aged 3 to 76 in North China showed that panuveitis uveitis (68/127, 53.5%) was the most common anatomical presentation among pediatric patients, followed by anterior (53/127, 41.7%), posterior (5/127, 4.0%) and intermediate (1/127, 0.8%), the composition of which was different from that of our cohort [30]. This discrepancy may be due to the difference of hospital and city, and the different subjective opinions for anatomical classification criterion. However, the etiologies in this study were consistent with our result.

The presence of serum ANA appeared to be a risk factor for uveitis in JIA patients [31]. However, it was not helpful in predicting the timing or severity of this comorbidity [32], and false positivity and transient positivity of the ANA (e.g. secondary to infections) were common occurrences. Therefore, it is recommended that patients with certain subtypes of JIA and positive ANA have more frequent screening eye examinations [33]. In Turkey, HLA-B27-associated uveitis accounted for 3.9% to 6.3% of all uveitis cases [34]. The incidence of acute anterior uveitis (AAU) in HLA-B27 positive patients as revealed in a meta-analysis varied from 40% to 82.5% [35]. In our pediatric cohort, HLA-B27-related uveitis accounted for 1.9% of 209 patients (four with idiopathic intermediate uveitis). Patients referred to our tertiary referral center may have more severe disease conditions, which may lead to a low rate of HLA-B27-related AAU. Serological molecular mimicry exists between group A streptococcal antigens and retinal S antigen, which may explain the involvement of the eye during a host’s immunological reaction to the bacterium. Curragh et al. [36] reported that a type of ASO or anti-deoxyribonuclease-positive uveitis presented with a seasonal preponderance, typically presenting in winter or spring, is called post-streptococcal syndrome (PSUS). Herein, 73% of 11 Caucasian pediatric patients presented in the spring or winter months. Among our ten ASO positive patients, uveitis occurred in spring or winter in six patients (60%), which is inconsistent with the above study.

In total, 41.0% children were diagnosed with SIOHT. The reported incidence of steroid responsiveness among children varies considerably between 3 and 87%, which may be explained by the heterogeneity of study designs, definition of steroid responsiveness, and differences in age and ethnicity of the populations included [37]. Infants and children under ten years of age might have a marked IOP response to corticosteroids [38]. Young age is a considerable risk factor for severe steroid-induced ocular hypertension, probably due to immaturity of the trabecular meshwork [37]. We found no significant difference between intermediate and high responders in sex, age, or steroid use duration, but the daily dose of non-responders was significantly lower than that of responders, indicating that the children with frequent instillation of eyedrops were at high risk, and the potential ratio of responders among children should be higher than that we found. It is interesting that a higher ratio of high responders (66.7%) was found within 14 days after topical corticosteroids were applied, suggesting that innate genetic susceptibility is likely important in the development of SIOHT. Some studies have attempted to explain this genetic susceptibility by a monozygotic autosomal mechanism. Medium responders may be heterozygous, while high responders are homozygous [38]. The time frame when ocular hypertension begins depends on the specific drug, dosage, frequency, route of administration, and susceptibility of the individual patient [38]. In our patients, IOP increase ranged from 7 to 259 days after initiating topical steroid use. The direct mechanism for SIOHT may be due to increased barrier function at the inner wall of Schlemm’s canal, alterations in cell contractility or extracellular matrix (ECM) turnover in the trabecular meshwork [39]. Therefore, for children who use corticosteroids, especially the local application of corticosteroids, IOP should be monitored closely and regularly to prevent irreversible optic nerve damage.

Cataracts are one of the most common and visually debilitating complications of pediatric uveitis. It develops as a consequence of chronic inflammation and corticosteroid use [40]. Eyes with pre-existing inflammation have a compromised blood-aqueous barrier, which is responsible for an even greater postoperative disruption of the barrier, leading to fibrin formation and consequent development of inflammatory sequelae [6]. Due to the lack of studies that show good evidence in terms of preoperative management, there is no consensus on the ideal treatment for cataract surgery in eyes with uveitis. We provided strict preoperative management in our cohort: ‘zero cells’ in the anterior chamber are the most important surgery indication, similar to a study by Rohl et al., which reported that a quiescence period of at least 30 days was beneficial in reducing uveitis recurrence after cataract surgery in adult patients [41]. Although most uveitis specialists suggest increasing corticosteroid treatment in the perioperative period among patients with chronic uveitis [36], in our cohort, patients continued to be slowly tapered preoperatively, while only adjuvant CT increased shortly before surgery. There appears to be a concern that IOL may trigger intraocular inflammation in uveitic eyes in children [6]. Previously, surgeons suggested keeping eyes aphakic for better visual outcomes [42, 43]. Gradually changing attitudes and better postoperative results have been reported in recently published studies and are attributed to improved medical control of inflammation, new surgical techniques, and more biocompatible IOLs [6]. Guindolet et al. and O'Rourke et al. noted that cataract surgery with primary posterior chamber hydrophobic IOL implantation is possible and leads to good visual recovery in pediatric chronic-uveitis cases [44, 45]. Based on strict inflammation control, we implanted IOLs for all patients and found no IOL-related aggravation of inflammation, and all patients were satisfied with the visual outcome and long-term remission of inflammation.

To our knowledge, this is the first report to comprehensively describe the pattern, treatment and therapeutic outcomes of childhood uveitis in China. Most children require long-term immunosuppressive treatment and the introduction of biological therapies due to their chronic course. An appropriate therapeutic regime based on accurate classification diagnosis, inflammation evaluation, and good management of complications are important for long-term prognosis. The patient prognosis was generally good after treatment and regular follow-up; however, most vision-treating complications occurred before referral to our hospital, indicating that the diagnosis and treatment of pediatric uveitis is a great challenge in primary hospitals in China. A prospective multicenter study would contribute to further delineating the disease types and clinical features of pediatric uveitis in China.

## Conclusion

In the current research, panuveitis, anterior uveitis, and intermediate uveitis were the most common presentations. JIA was the most common associated systemic disease. Most cases were of chronic uveitis and scleritis requiring long-term systemic immunosuppressive therapies. Good management of complications is important for long-term prognosis.

## Data Availability

The ethics committee of the Tianjin Medical University Eye Hospital approved collection of clinical data for this retrospective study. In order to protect patient anonymity, the clinical data used and analyzed in the current study are not all publicly available but are available on reasonable request from the corresponding author.
